# Conserved gene signatures shared among *MAPT* mutations reveal defects in calcium signaling

**DOI:** 10.3389/fmolb.2023.1051494

**Published:** 2023-02-09

**Authors:** Miguel A. Minaya, Sidhartha Mahali, Abhirami K. Iyer, Abdallah M. Eteleeb, Rita Martinez, Guangming Huang, John Budde, Sally Temple, Alissa L. Nana, William W. Seeley, Salvatore Spina, Lea T. Grinberg, Oscar Harari, Celeste M. Karch

**Affiliations:** ^1^ Department of Psychiatry, Washington University in St Louis, St Louis, MO, United States; ^2^ Neural Stem Cell Institute, Rensselaer, NY, United States; ^3^ Department of Neurology, UCSF Weill Institute for Neurosciences, University of California, San Francisco, San Francisco, CA, United States; ^4^ Department of Pathology, University of Sao Paulo, Sao Paulo, Brazil; ^5^ Hope Center for Neurological Disorders, Washington University in St Louis, St Louis, MO, United States; ^6^ NeuroGenomics and Informatics Center, Washington University in St Louis, St Louis, MO, United States

**Keywords:** IPSC-derived neurons, frontotemporal dementia (FTD), MAPT mutations (tau), transcriptomics, calcium signaling

## Abstract

**Introduction:** More than 50 mutations in the *MAPT* gene result in heterogeneous forms of frontotemporal lobar dementia with tau inclusions (FTLD-Tau). However, early pathogenic events that lead to disease and the degree to which they are common across *MAPT* mutations remain poorly understood. The goal of this study is to determine whether there is a common molecular signature of FTLD-Tau.

**Methods:** We analyzed genes differentially expressed in induced pluripotent stem cell–derived neurons (iPSC-neurons) that represent the three major categories of *MAPT* mutations: splicing (IVS10 + 16), exon 10 (p.P301L), and C-terminal (p.R406W) compared with isogenic controls. The genes that were commonly differentially expressed in *MAPT* IVS10 + 16, p.P301L, and p.R406W neurons were enriched in trans-synaptic signaling, neuronal processes, and lysosomal function. Many of these pathways are sensitive to disruptions in calcium homeostasis. One gene, *CALB1*, was significantly reduced across the three *MAPT* mutant iPSC-neurons and in a mouse model of tau accumulation. We observed a significant reduction in calcium levels in *MAPT* mutant neurons compared with isogenic controls, pointing to a functional consequence of this disrupted gene expression. Finally, a subset of genes commonly differentially expressed across *MAPT* mutations were also dysregulated in brains from *MAPT* mutation carriers and to a lesser extent in brains from sporadic Alzheimer disease and progressive supranuclear palsy, suggesting that molecular signatures relevant to genetic and sporadic forms of tauopathy are captured in a dish. The results from this study demonstrate that iPSC-neurons capture molecular processes that occur in human brains and can be used to pinpoint common molecular pathways involving synaptic and lysosomal function and neuronal development, which may be regulated by disruptions in calcium homeostasis.

## Background

Frontotemporal lobal degeneration with tau inclusions (FTLD-tau) encompasses a heterogenous group of disorders characterized by frontal and temporal lobar atrophy, neuronal loss and gliosis, and the accumulation of neurofibrillary tangles (NFTs) (Bodea et al., 2016). In a subset of cases, FTLD-Tau is caused by rare, dominantly inherited mutations in the microtubule associated protein tau (*MAPT*) gene (Pottier et al., 2016). Common genetic variation in the *MAPT* gene also contributes to sporadic forms of FTLD-Tau, including progressive supranuclear palsy (PSP) and corticobasal degeneration (Ferrari et al., 2014; Kouri et al., 2015; Steele et al., 2018).

*MAPT* is alternatively spliced and developmentally regulated in the central nervous system, resulting in six canonical tau isoforms, which differ based on the absence (0N) or presence of one or two N-terminal inclusions (1N, 2N, respectively) and three or four repeats in the microtubule binding region (3R or 4R, respectively) (Neve et al., 1986; Lee et al., 2001). In adult brains, there is an equal balance of 3R and 4R tau isoforms. More than 50 mutations in the *MAPT* gene are reported to cause FTLD-Tau (https://www.alzforum.org/mutations/mapt). These mutations fall into three major categories. First, located in the intronic region near the stem-loop domain, a subset of mutations alter *MAPT* splicing *via* inclusion of exon 10 (e.g., 3R<4R tau) or exclusion of exon 10 (e.g., 3R>4R tau). Second, missense mutations may occur within exon 10, such that the mutation is only present in a subset of *MAPT* isoforms (i.e. 4R tau). Finally, missense mutations may occur outside of the microtubule binding region, which leads to the production of mutant protein among all tau isoforms. We asked whether the heterogeneity in *MAPT* mutations drive common molecular mechanisms. To begin to address this question, we studied *MAPT* mutations that fall into these three major categories: *MAPT* IVS10 + 16, p.P301L and p.R406W, respectively.

Human cellular models of the brain derived from induced pluripotent stem cells (iPSC) have become an important tool for studying molecular and cellular markers that may initiate disease (Livesey, 2014; Iovino et al., 2015; Silva et al., 2016; Guo et al., 2017; Wray, 2017; Gonzalez et al., 2018; Jiang et al., 2018; Karch et al., 2018; Nakamura et al., 2019; Bowles et al., 2021; Lagomarsino et al., 2021). Here, we coupled human iPSC models with CRISPR/Cas9 genome editing technology to create a system that allows us to distinguish the molecular signatures associated with *MAPT* mutations and to begin to resolve the molecular phenotypes of tauopathy. Together, our findings uncover key changes in trans-synaptic signaling, lysosomal function, and calcium signaling shared across *MAPT* mutations.

## Materials and methods

### Patient consent

Skin punches were performed following written informed consent from the donor. The informed consent was approved by the Washington University School of Medicine and University of California San Francisco Institutional Review Board and Ethics Committee (IRB 201104178, 201306108 and 10-03946). The consent allows for use of tissue by all parties, commercial and academic, for the purposes of research but not for use in human therapy.

The Washington University and University of California San Francisco Institutional Review Boards reviewed the Neuropathology Cores (from whom the brains were obtained) operating protocols as well as this specific study and determined it was exempt from approval. Our participants provide this consent by signing the hospital’s autopsy form. If the participant does not provide future consent before death the DPOA or next of kin provide it after death. All data were analyzed anonymously.

### iPSC lines

Human iPSC used in this study (Supplementary Table S1; Supplementary Figure S1) have been previously described (Karch et al., 2019). Briefly, iPSC lines were generated using non-integrating Sendai virus carrying OCT3/4, SOX2, KLF4, and cMYC (Life Technologies) (Takahashi and Yamanaka, 2006; Ban et al., 2011). iPSC lines were characterized for the following parameters using standard methods (Takahashi and Yamanaka, 2006): pluripotency markers by immunocytochemistry (ICC) and quantitative PCR (qPCR), spontaneous or TriDiff differentiation into the three germ layers by ICC and qPCR, assessment of chromosomal abnormalities by karyotyping, and *MAPT* mutation status was confirmed by Sanger sequencing (Supplementary Figure S1). To determine the impact of the *MAPT* mutant allele on molecular phenotypes, we used CRISPR/Cas9-edited isogenic controls in which the mutant allele was reverted to the wild-type (WT) allele in each of the donor iPSC lines as previously described (GIH36C2; F11362.1; F0510.2; Supplementary Table S1; Supplementary Figure S1) (Karch et al., 2019). Resulting edited lines were characterized as described above in addition to on- and off-target sequencing (Supplementary Figure S1). All iPSC lines used in this study carry the *MAPT* H1/H1 common haplotype.

### Differentiation of iPSCs into cortical neurons

iPSCs were differentiated into cortical neurons using a two-step approach as previously described (Karch et al., 2019) (https://dx.doi.org/10.17504/protocols.io.p9kdr4w). iPSCs were plated at a density of 65,000 cells per well in neural induction media (StemCell Technologies) in a 96-well v-bottom plate to form neural aggregates and after 5 days, transferred into culture plates. The resulting neural rosettes were then isolated by enzymatic selection (Neural Rosette Selection Reagent; StemCell Technologies) and cultured as neural progenitor cells (NPCs). NPCs were differentiated in planar culture in neuronal maturation medium (neurobasal medium supplemented with B27, GDNF, BDNF, cAMP). Neurons typically arose within 1 week after plating, identified using immunocytochemistry for β-tubulin III (Tuj1). The cells continue to mature and were analyzed at 6 weeks.

### RNA extraction, sequencing, and transcript quantification

iPSC-derived neurons were re-suspended in 200 µL of 50:1 homogenization solution: 1-Thioglycerol solution. After addition of 200 µL of Promega lysis buffer, the samples were transferred to the appropriate well of the Maxwell RSC cartridge. DNase solution was added to each cartridge. TapeStation 4200 System (Agilent Technologies) was used to perform quality control of the RNA concentration, purity, and degradation based on the estimated RNA integrity Number (RIN), and DV200 (Supplementary Table S1). Samples were sequenced by an Illumina HiSeq 4000 Systems Technology with a read length of 1 × 150 bp, and an average library size of 36.5 ± 12.2 million reads per sample.

Identity-by-Descent (IDB) (Browning and Browning, 2010) and FastQC (Andrews et al., 2012) analyses were performed to confirm sample identity. STAR (v.2.6.0) (Dobin et al., 2012) was used to align the RNA sequences to the human reference genome: GRCh38.p13 (hg38). The quality of RNA alignment was evaluated using sequencing metrics such as read distribution, ribosomal content, and alignment quality in Picard (v.2.8.2). The average percentage of unique mapped reads in the BAM files was 80.3% ± 3.62, and the average percentage of total mapped reads to GRCh38.p13 was 90.1% ± 5.12 (Supplementary Table S1). IGV (Integrative Genomics Viewer) (Thorvaldsdottir et al., 2013) was used with the reference Human Genome (hg38) to visualize mutation containing reads and their absence in samples edited using CRISPR/Cas9 protocols (isogenic controls).

Salmon (v. 0.11.3) (Patro et al., 2017) was used to quantify the expression of the genes annotated within the human reference genome used in this project (GRCh38.p13). Protein coding genes were selected for downstream analyses.

### Principal component and differential expression analyses

Principal component analyses (PCA) were performed based on 19,957 protein coding genes using regularized-logarithm transformation (rlog) counts. Differential gene expression was performed using the DESeq2 (v.1.22.2) R package (Love et al., 2014). PCA and differential gene expression analyses were performed independently for each set of *MAPT* mutations and isogenic controls. Each *MAPT* mutation and its isogenic control were considered independent cohorts due to their shared genetic background. As such, the relationship across the three *MAPT* mutation sets was evaluated using the *MetaVolcanoR* R package (v1.10.0) (Prada et al., 2021). The meta-analysis included those genes that were differentially expressed (*p* <0.05) in the same direction across the three cohorts (*n* = 275 genes). A meta-volcano plot summarizing the gene fold change of the *MAPT* IVS10 + 16, p.P301L, and p.R406W datasets was generated using a Random Effect Model (REM) estimation. PCA and Volcano plots were created for each comparison using the ggplot2 R package (v3.3.6) (Wickham, 2016).

### Pathway enrichment and network analyses

ToppGene (Chen et al., 2009) and Enrichr (Chen et al., 2013; Kuleshov et al., 2016; Xie et al., 2021) were used to identify pathways in which differentially expressed genes are enriched. Gene ontologies (GOs) related to molecular function, biological process and cellular component were selected based on two criteria: i) *p* ≤ 0.05 and ii) number of query genes associated with each GO > 1. Gene relationships including physical, predicted and genetic interactions, and gene networks including co-expression and co-localization were annotated using the geneMANIA prediction server (Warde-Farley et al., 2010).

### Mouse model of tauopathy

To evaluate whether the genes differentially expressed in iPSC-derived neurons were altered in animal models of tauopathy, we analyzed the gene expression in the Tau-P301L mouse model of tauopathy and non-transgenic controls (Ramsden et al., 2005). Transcriptomic data from mice was obtained from the Mouse Dementia Network (Matarin et al., 2015). Gene expression across the timepoints (2-, 4-, 8-, and 18-months old mice) was normalized to mice at 2 months of age and plotted. Differential gene expression at 18 months of age was analyzed by unpaired t-tests to assess significance.

### Drug target identification

To determine whether differentially expressed genes were associated with known drugs, we interrogated: (i) the WEB-based Gene SeT AnaLysis Toolkit (Liao et al., 2019), (ii) the Drug-Gene Interaction Database (Freshour et al., 2021), and (iii) the DrugBank (Wishart et al., 2018).

### Calcium imaging

To measure calcium levels in iPSC-derived neurons, *MAPT* IVS10 + 16 mutation (GIH36C2) and isogenic controls (GIH36C2Δ1D01) were analyzed. NPCs were differentiated into cortical neurons as described above. After 22 days in culture, 2 × 10^5^ neurons of each genotype were seeded into poly-L-ornithine and laminin-coated 96-well plate. Ca^2+^ levels in the iPSC-derived neurons were then measured using the Invitrogen™ Fluo-4 Direct™ Calcium Assay Kit (catalog number: F10471) following manufacturer’s instructions. Briefly, at 36 days in culture, growth medium was replaced with 50 μL per well Fluo-4 Direct^TM^ calcium assay buffer and 50 μL per well of the 2x Fluo-4 Direct^TM^ calcium reagent loading solution. The 96-well plate was then incubated at 37°C for 60 min, after which Fluo-4 fluorescence in intact cells directly proportional to cytoplasmic Ca^2+^ levels was measured using Synergy HTX multi-mode microplate reader (BioTek Instruments excitation at 494 nm and emission at 516 nm). Negative controls included Fluo-4 Direct^TM^ calcium assay buffer plus reagent with no neurons and neurons without assay reagent. Fluo-4 staining in cells was imaged under a Nikon Eclipse 80i fluorescent microscope at 20x magnification. After measuring cytoplasmic Ca^2+^ levels, cells were lysed to break cellular and organelle membranes in 1% Triton X-100 and total Fluo-4 fluorescence intensities from cytoplasmic and intracellular Ca^2+^ stores were measured as described above.

### Human brain datasets

To determine whether the differentially expressed genes in the iPSC-derived neurons capture molecular processes that occur specifically in primary tauopathies or that represent more general pathways associated with neurodegeneration, we analyzed gene expression in human brains with primary tauopathy (e.g., *MAPT* mutation carriers and progressive supranuclear palsy (PSP)), secondary tauopathy (e.g., Alzheimer disease (AD)), and FTLD with TDP-43 pathology (FTLD-TDP). Primary tauopathy datasets included: i) middle temporal gyrus from *MAPT* IVS10 + 16 mutation carriers (2 samples) and healthy controls (3 samples); ii) insular cortex from *MAPT* R406W carriers (2 samples) and healthy controls (2 samples) (Jiang et al., 2018); and iii) Temporal cortex from progressive supranuclear palsy (PSP) brains (82 samples) and healthy control brains (76 samples; syn6090813) (Allen et al., 2015; Allen et al., 2016). Secondary tauopathy datasets included temporal cortex from AD brains (84 samples) and healthy controls (76 samples) (Allen et al., 2015; Allen et al., 2016). To determine whether gene expression changes in iPSC-neuron models reflect a more general impact on neurodegenerative pathways, we examined gene expression profiles isolated from tissue of FTLD-TDP caused by rare mutations the *GRN*, *C9ORF72* expansions, or from sporadic cases (Knight ADRC)(Li et al., 2018; Dube et al., 2019; Wani et al., 2021): i) parietal lobe from *GRN* mutation carriers (5 samples), ii) *C9ORF72* expansion carriers (5 samples), iii) sporadic cases (8 samples), and (iv) healthy controls (16 samples). Differential gene expression analyses comparing controls and disease diagnosed brains were performed using gene expression measures and including as covariates sex, age-at-death, RNA integrity number (RIN), and brain tissue source.

## Results

### MAPT mutations are sufficient to induce global transcriptomic changes in human neurons

**FIGURE 1:**
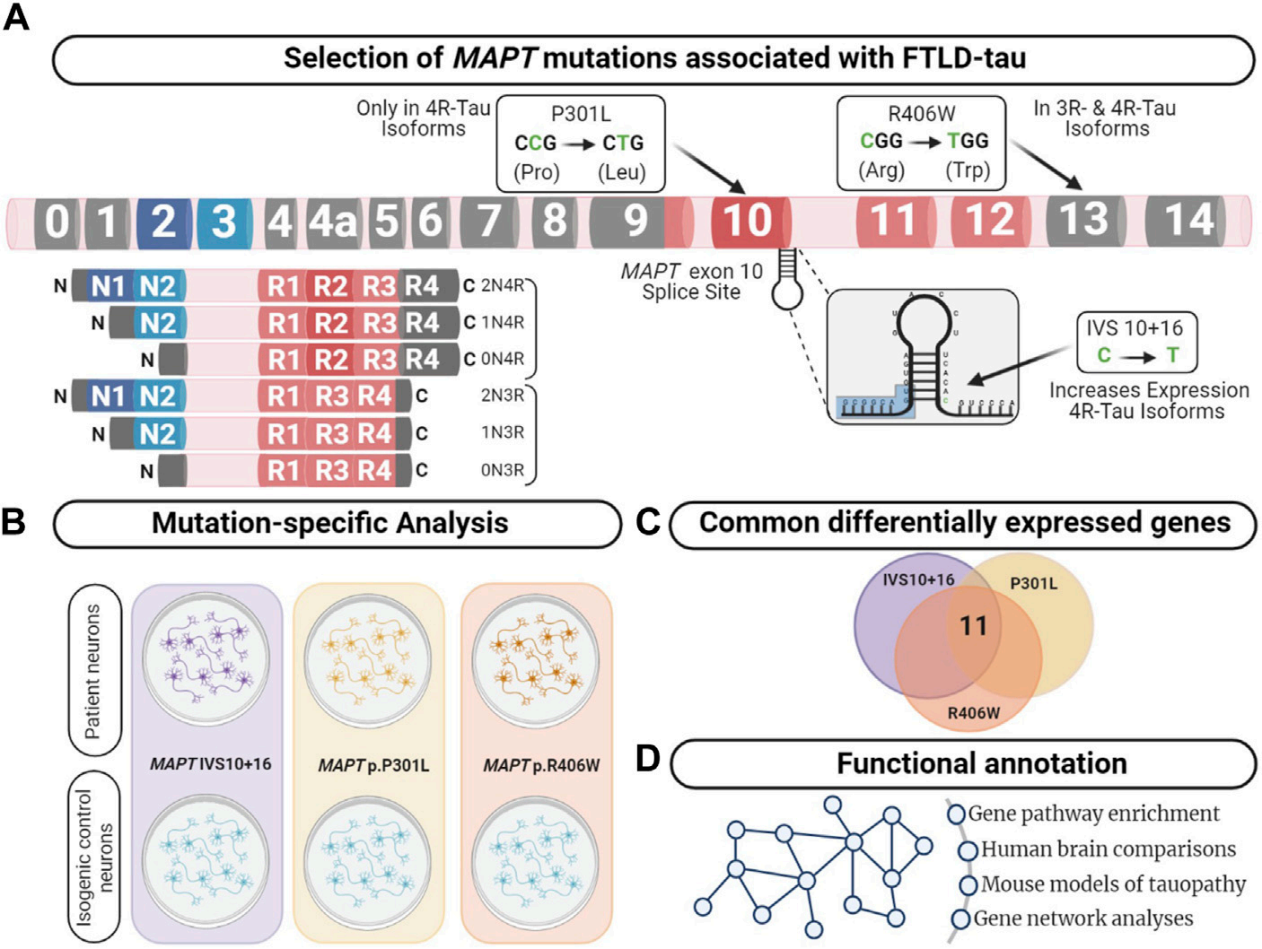
Integrative analysis to identify dysregulated pathways in FTLD-Tau. **(A)** *MAPT* gene annotated with the location of the mutations used in this study. Lower left panel displays the six major isoforms expressed in the central nervous system. **(B)** Comparison of human iPSC-neurons carrying the *MAPT* mutation specific and isogenic controls served as a discovery cohort to identify genes dysregulated across the three mutations. **(C)** Overlap analysis between all mutants compared with control. After multiple test corrections (BY-FDR ≤ 0.05), we identified 11 commonly differentially expressed genes. **(D)** Functional annotation was performed using the commonly differentially expressed genes.

**FIGURE 2:**
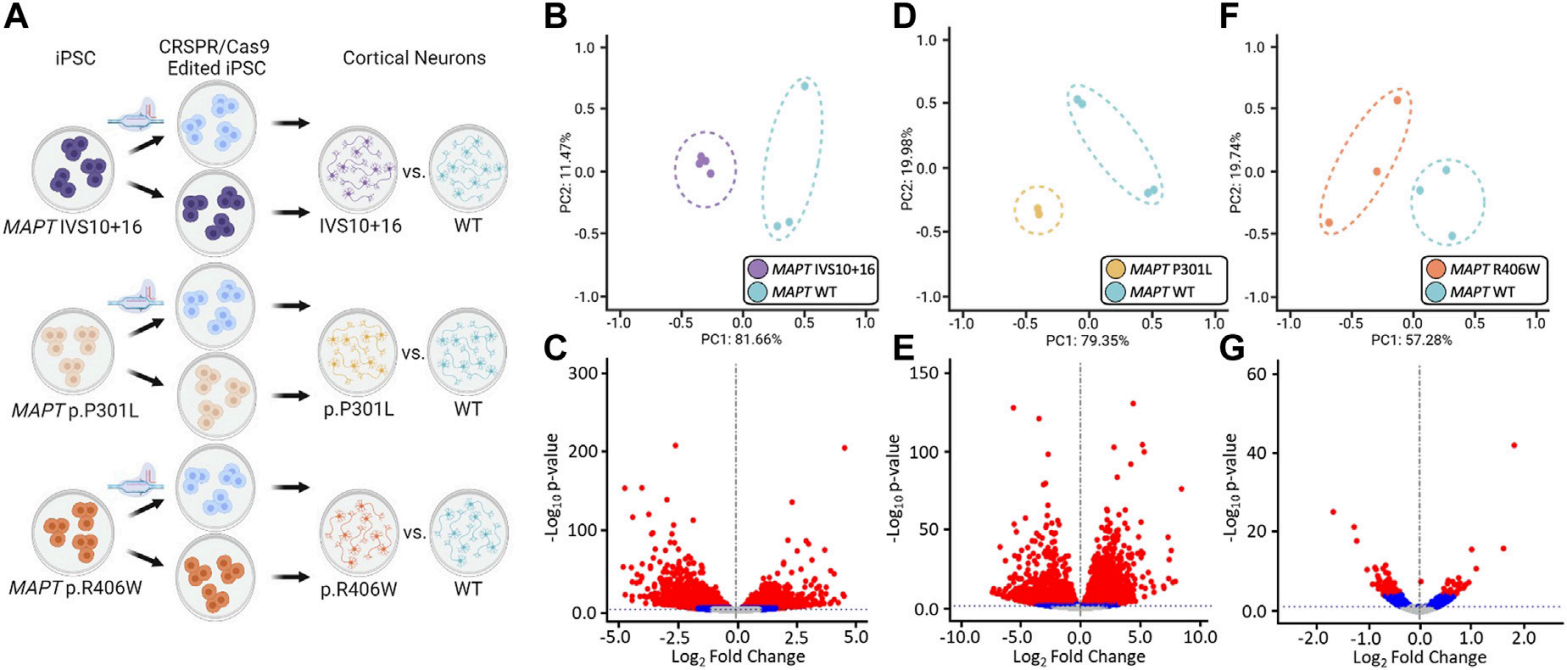
Global transcriptomic effects of *MAPT* IVS10 + 16, p.P301L, and p.R406W mutations. **(A)** Overview of iPSC differentiation into cortical neurons. **(B–C)** Principal component analyses and volcano plots obtained from *MAPT* IVS10 + 16 neurons compared to isogenic controls. **(D–E)** PCA and Volcano plots obtained from *MAPT* p.P301L neurons compared to isogenic controls. **(F–G)** PCA and Volcano plots obtained from *MAPT* p.R406W neurons compared to isogenic controls. PCA and Volcano plots were based on 19,957 protein-coding genes using regularized-logarithm transformation (rlog) counts. Volcano plots showing log_2_fold change between iPSC-derived neurons carrying *MAPT* mutations vs. isogenic controls, and the –log_10_ *p*-value for each gene. Red and blue dots within volcano plots represent, respectively, genes differentially expressed under adjusted-BY (BY-FDR) and unadjusted *p*-values (*p* ≤ 0.05).

The goal of this study was to identify common genes and pathways that are downstream of *MAPT* mutations and candidate drivers of disease pathogenesis in FTLD-Tau (Figure 1). To address this goal, we studied a series of *MAPT* mutations that represent three major mutation types: *MAPT* IVS10 + 16, p.P301L and p.R406W (Figure 1; Supplementary Table S1). Protein coding genes obtained from RNA-sequencing data generated from iPSC–derived neurons carrying one of these three *MAPT* mutations together with isogenic controls were analyzed (Figure 2A). Among isogenic pairs, each of the *MAPT* mutations were sufficient to induce global transcriptomic changes in iPSC-neurons: 81.66% principal component 1 (PC1) for *MAPT* IVS10 + 16; 79.33% PC1 for *MAPT* p.P301L; and 57.28% PC1 for *MAPT* p.R406W (Figures 2B,D,F). PCA of the CRISPR/Cas9-engineered *MAPT* WT lines from independent donors reveal donor-dependent clustering (Supplementary Figure S2), suggesting that genetic background of the donor is the largest driver of transcriptomic variation which is consistent with prior reports (Kilpinen et al., 2017). Given that the genetic background remains conserved within the isogenic pairs, we treated each pair as a cohort and performed differential expression analyses to determine the impact of the presence of each mutant allele (Figures 2C,E,G; Supplemental Tables 2 and 3). Together, these findings illustrate that FTLD-causing *MAPT* mutations are sufficient to produce robust gene expression changes in neurons.

### MAPT mutations produce a shared gene expression signature in human neurons

**FIGURE 3:**
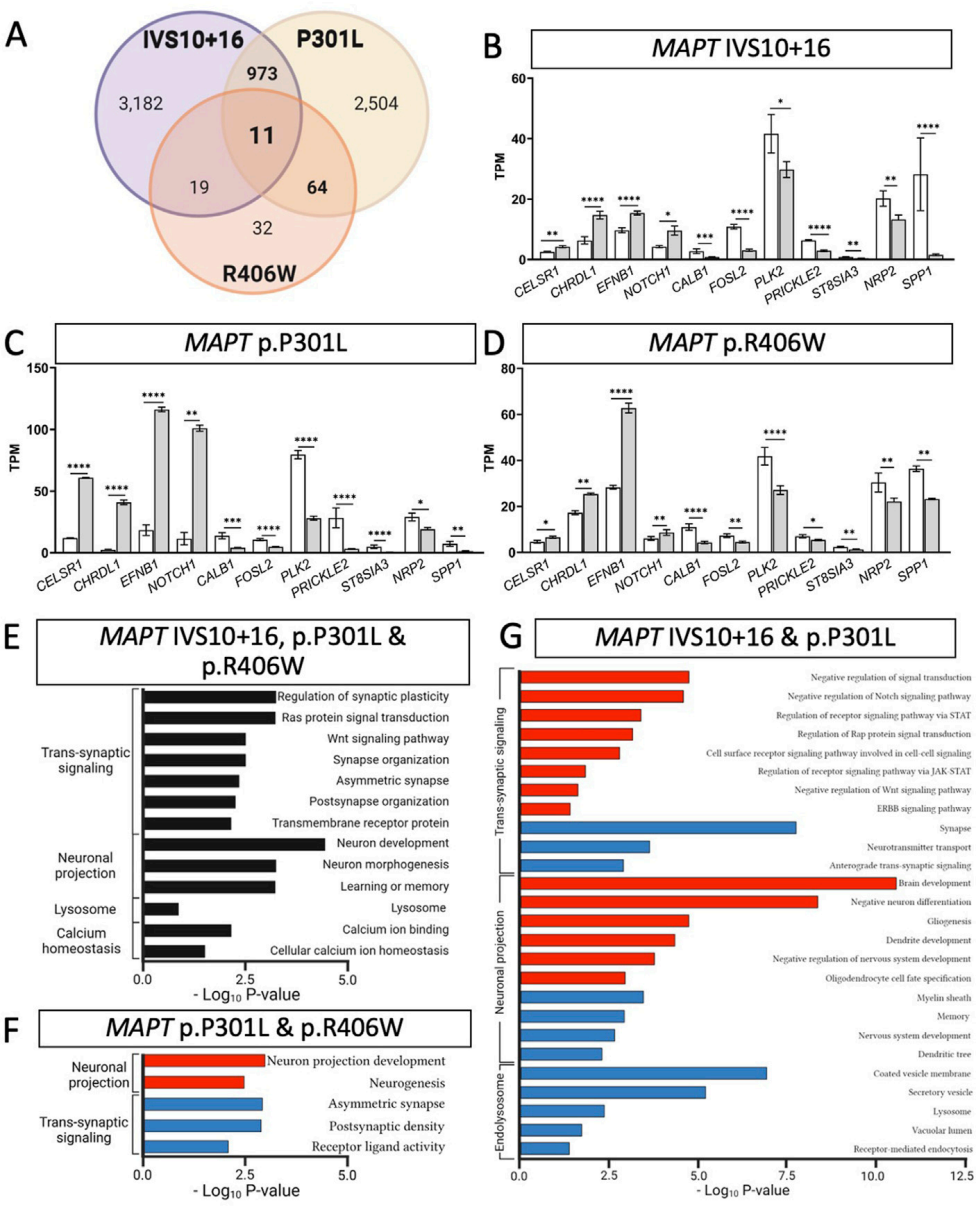
*MAPT* mutations result in common defects in synaptic signaling, neuronal projection, and lysosomal function. **(A)** Venn diagram presenting the differentially expressed genes common among iPSC-neurons carrying *MAPT* IVS10 + 16, p.P301L and p.R406W mutations (BY-FDR ≤ 0.05). **(B–D)** Normalized TPM expression of 11 genes shared between the three datasets (*MAPT-*IVS10 + 16, p.P301L and p.R406W; BY-FDR ≤ 0.05). **(E)** Bar graph showing the most significant pathways enriched among the 11 shared differentially genes (black bars). **(F)** Bar graph of the pathways enriched among the genes shared between *MAPT* p.P301L and p.R406W neurons. **(G)** Bar graph of the pathways enriched among the genes shared between *MAPT* IVS10 + 16 and p.P301L neurons. **(F–G)** Pathways from up-regulated genes (red bars). Pathways from down-regulated genes (blue bars).

Differential gene expression analyses within isogenic pairs illustrates that individual *MAPT* mutations produce global transcriptomic changes; thus, we sought to determine the extent of overlap in the differentially expressed genes among these three distinct *MAPT* mutation types (Figure 1C). We identified 11 differentially expressed genes across the three datasets (BY-FDR<0.05) (Figures 3A–D; Supplementary Table S4): *CELSR1*, *CHRDL1*, *EFNB1*, *NOTCH1*, *CALB1*, *FOSL2*, *PLK2*, *PRICKLE2*, *ST8SIA3*, *NRP2*, *SPP1*. Pathway analyses revealed that the 11 genes were enriched for i) trans-synaptic signaling pathways; ii) neuronal projection pathways; iii) lysosomal functions; and iv) calcium homeostasis (Figure 3E). These findings point to a common set of genes and pathways that are altered downstream of three distinct classes of *MAPT* mutations.

### Unique gene signatures

Beyond molecular signatures shared across the three *MAPT* mutations, we observed an imbalance in those genes shared between point mutations (*MAPT* p.P301L and p.R406W) and exon 10 mutations (*MAPT* IVS10 + 16 and p.P301L). We identified 64 genes that were shared between iPSC-neurons carrying the *MAPT* p.P301L and p.R406W mutations (BY-FDR<0.05; Figure 3A; Supplementary Table S5), while 973 genes were shared between iPSC-neurons carrying the *MAPT* IVS10 + 16 and p.P301L mutations (BY-FDR<0.05; Figure 3A; Supplementary Table S6). Thus, the number of differentially expressed genes associated with mutations located around the alternatively spliced exon 10 was 15-fold higher than the number of dysregulated genes associated with the *MAPT* p.P301L and p.R406W mutations.

Despite the different patterns in gene expression, pathway analyses were consistent with observations across all three *MAPT* mutations. The 64 dysregulated genes shared between the *MAPT* p.P301L and p.R406W neurons were enriched in pathways related to neurogenesis and trans-synaptic signaling (Figure 3F; Supplementary Table S7). Among *MAPT* IVS 10 + 16 and p.P301L (*n* = 973 genes), 409 up-regulated genes were enriched in pathways involved in the regulation of cell signaling: i) receptor signaling *via* JAK-STAT pathway; ii) Rap protein signal transduction; and iii) ERBB signaling pathway (Figure 3G; Supplementary Table S8). The 564 down-regulated genes were enriched in pathways involved in endolysosomal function: i) the coated vesicle membrane; ii) secretory vesicles; iii) lysosome; and iv) vacuolar lumen (Figure 3G; Supplementary Table S8).

A number of genes were found to be uniquely differentially expressed within each of the isogenic pairs, suggesting mutation-specific effects on gene expression (Figure 3A; Supplementary Table S3). The *MAPT* IVS10 + 16 mutation led to a significant increase in 1,804 unique genes, which are associated with synapse, learning, and vesicle-mediated transport (Supplementary Table S9), while the 1,746 uniquely down-regulated genes were associated with lysosome functions and apoptotic signaling (Supplementary Table S9). The *MAPT* p.P301L mutation produced 1,333 unique up-regulated genes that were associated with cell cycle processes (e.g., mitosis, meiosis, organellar fusion/division) and 1,645 unique down-regulated genes were associated with vesicle-mediated transport and neuron projections (Supplementary Table S9). Finally, the *MAPT* p.R406W mutation resulted in 217 uniquely up-regulated genes that were associated with lysosomal pathways, and the 174 uniquely down-regulated genes were associated with GABA receptor complex, neurotransmitter receptor activities, and synaptic signaling (Supplementary Table S9). Together, we demonstrate that many of these uniquely differentially expressed genes fall within pathways shared among all the *MAPT* mutations.

### MAPT mutations lead to common genetic signatures that are associated with tau aggregation in mouse models of tauopathy

**FIGURE 4:**
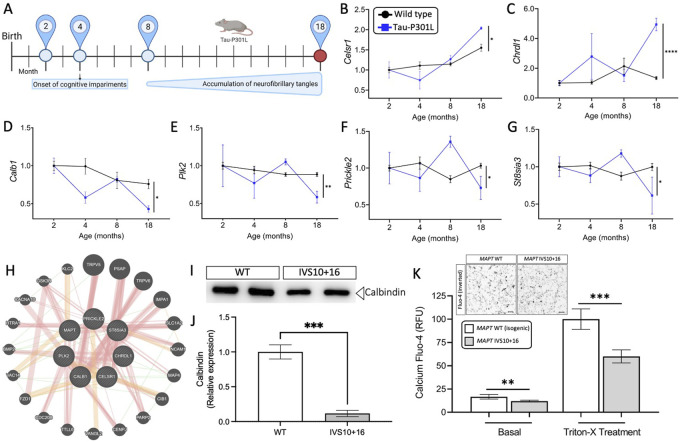
Genes altered in iPSC-neurons from *MAPT* mutation carriers are replicated in disease end-stage in the Tau-P301L tauopathy mouse model. **(A)** The expression of the 11 genes observed in iPSC-neurons were evaluated in the mouse model of tauopathy (Tau-P301L vs. wild type). Statistical comparisons reflect gene expression at disease end-stage (18 months old mice). **(B–G)** Expression of the six selected genes (2 up- and 4 down-regulated) in mice. Black circles, transgenic Tau-P301L mice; blue squares, non-transgenic control mice. Graphs show normalized gene expression relative to 2 months old mice. The genes presented were selected based on: **(I)** the expression of the genes in the human cell models and in mice followed the same direction, and (ii) the difference in the expression of each gene in 18-month old controls and transgenic Tau-P301L mice was significant. Statistical analyses (t-tests) were based on values normalized related to mice that were 2 months old. *p ≤ 0.05; **p ≤ 0.01; ****p ≤ 0.0001. **(H)** Relatedness of the six selected genes dysregulated in human and mice, including the *MAPT* gene. The plot demonstrates the strong physical (orange nodes: 80.3%) and genetic interactions (green nodes 4.28%) between the query genes and genes have been related to pathways such as neurogenesis, behavior, learning or memory, and glutamatergic synapse. The size of the gene nodes is proportional to the degree to which the genes are related. Query genes are presented as striped dark grey balls and other selected genes subjected to interaction are presented as dark grey balls without stripes. Physical (orange; 80.3%), predicted (yellow; 9.48%), and genetic (green; 4.28%) interactions are displayed within the network. **(I–K)** Calcium is dysregulated in *MAPT* neurons. iPSC-neurons from *MAPT* IVS10 + 16 and isogenic control were cultured for 42 days and evaluated for calbindin 1 protein and intracellular calcium levels. **(I)** Immunoblot for calbindin 1 protein levels. **(J)** Quantification of calbindin 1 protein levels. **(K)** Inset, inverted fluorescent imaging representing the calcium concentration in iPSC-neurons treated with Fluo-4 Direct™ Calcium Assay Kit. Fluo4-Ca^2+^ binding dye relative fluorescence units (RFU) observed in iPSC-neurons carrying the IVS10 + 16 (grey bars) and isogenic controls (white bars) (*p ≤ 0.05; **p ≤ 0.01; ***p ≤ 0.001; ****p ≤ 0.0001). Scale bar 20uM.

We sought to determine the extent to which the 11 commonly differentially expressed genes across *MAPT* mutations (Figure 3) were altered during disease course in the Tau-P301L mouse model of tauopathy. Using the Mouse Dementia Network (Matarin et al., 2015), we analyzed transcriptomic data generated from the cortex of WT and Tau-P301L mice collected at 2, 4, 8, and 18-months (Figure 4A). Compared with WT littermates, transgenic Tau-P301L mice develop tau aggregates beginning at 8 months of age (Ramsden et al., 2005). Among the 11 genes, six genes were differentially expressed at 18 months of age, when tau aggregation is most prominent (Figures 4B–G; Supplementary Figure S3): *Celsr1*, *Chrdl1*, *Calb1*, *Plk2*, *Prickle2*, and *St8sia3*. These six genes are highly related to one another and enriched in pathways related to neurodegeneration such as neurogenesis, behavior, learning, memory, and glutamatergic synapse (Figure 4H; Supplementary Table S10). At earlier timepoints when tau aggregation is beginning in the Tau-P301L mouse model, we observed statistical differences in expression of *Efnb2* (8 months), *Fosl2* (8 months), *Calb1* (4 months), *Nrp2* (8 months), *Prickle2* (8 months), and *St8sia3* (8 months). The Drug-Gene Interaction Database (Freshour et al., 2021) and DrugBank (Wishart et al., 2018) revealed that two genes, *PLK2* and *CALB1*, are known targets of FDA approved drugs including tramadol, ethosuximide, levodopa, nicotine and oxcarbazepine, which are currently used to treat neurological symptoms (Supplementary Table S11). Together, these findings illustrate that *MAPT* mutations are sufficient to induce molecular changes in iPSC-neurons that are relevant to tau aggregation *in vivo*.

### Gene dysregulation downstream of *MAPT* mutations impact calcium content

Next, we sought to explore the functional consequences of *MAPT* mutation-driven gene changes. Pathways associated with trans-synaptic signaling and lysosomal function were commonly altered by the three *MAPT* mutations and are regulated by calcium signaling (Figure 3E) (Lloyd-Evans and Waller-Evans, 2020). Among the 11 commonly differentially expressed genes (Figure 3), *NOTCH1*, *PLK2*, *PRICKL2*, and *CALB1* are involved in calcium signaling. The *CALB1* gene, which encodes the calbindin 1 protein and regulates Ca^2+^ entry into cells upon the stimulation of glutamate receptors (Noble et al., 2018), was significantly down-regulated in *MAPT* mutant iPSC-neurons (Figures 3B–D) and in 18-month old Tau-P301L mice when tau aggregation was present (Figure 4D). Thus, we hypothesized that reduced *CALB1* expression leads to reduced intracellular calcium. To test this hypothesis, we verified that Calbindin 1 protein levels were significantly reduced in *MAPT* IVS10 + 16 neurons compared to isogenic controls (Figures 4I,J). We then measured calcium levels in iPSC-neurons from *MAPT* IVS10 + 16 and isogenic controls using Fluo-4 Direct^TM^, a cell-permeable fluorescent Ca^2+^ indicator. We observed a significant reduction in calcium levels under basal conditions, representing cytoplasmic calcium levels, in *MAPT IVS10+16* iPSC-neurons compared with isogenic controls (Figure 4K). After treating with Triton-X to release intracellular calcium stores, we found that total calcium levels were also significantly reduced in *MAPT IVS10+16* iPSC-neurons compared with isogenic controls (Figure 4K). Thus, we show that *MAPT* mutations are sufficient to disrupt calcium homeostasis in neurons.

### Stem cell models capture tauopathy-relevant gene signatures

Leveraging isogenic iPSC lines to understand the contribution of a single allele to downstream phenotypes is a powerful system that when applied here has revealed gene signatures shared across *MAPT* mutations that also change during tau accumulation in mouse models of tauopathy. However, a limitation of this approach remains that iPSC-neurons are cultured in a dish and remain relatively immature. For example, iPSC-neurons predominantly express 0N3R tau (Patani et al., 2012; Sposito et al., 2015; Sato et al., 2018), while the adult brain expresses six tau isoforms (Hefti et al., 2018; Sato et al., 2018). Thus, we next sought to determine the extent to which the gene signatures we observe in iPSC-neurons from *MAPT* mutations are relevant to gene expression changes occurring in human brains with tauopathy and the extent to which these gene signatures are occurring across neurodegenerative diseases (Supplementary Table S12).

**FIGURE 5:**
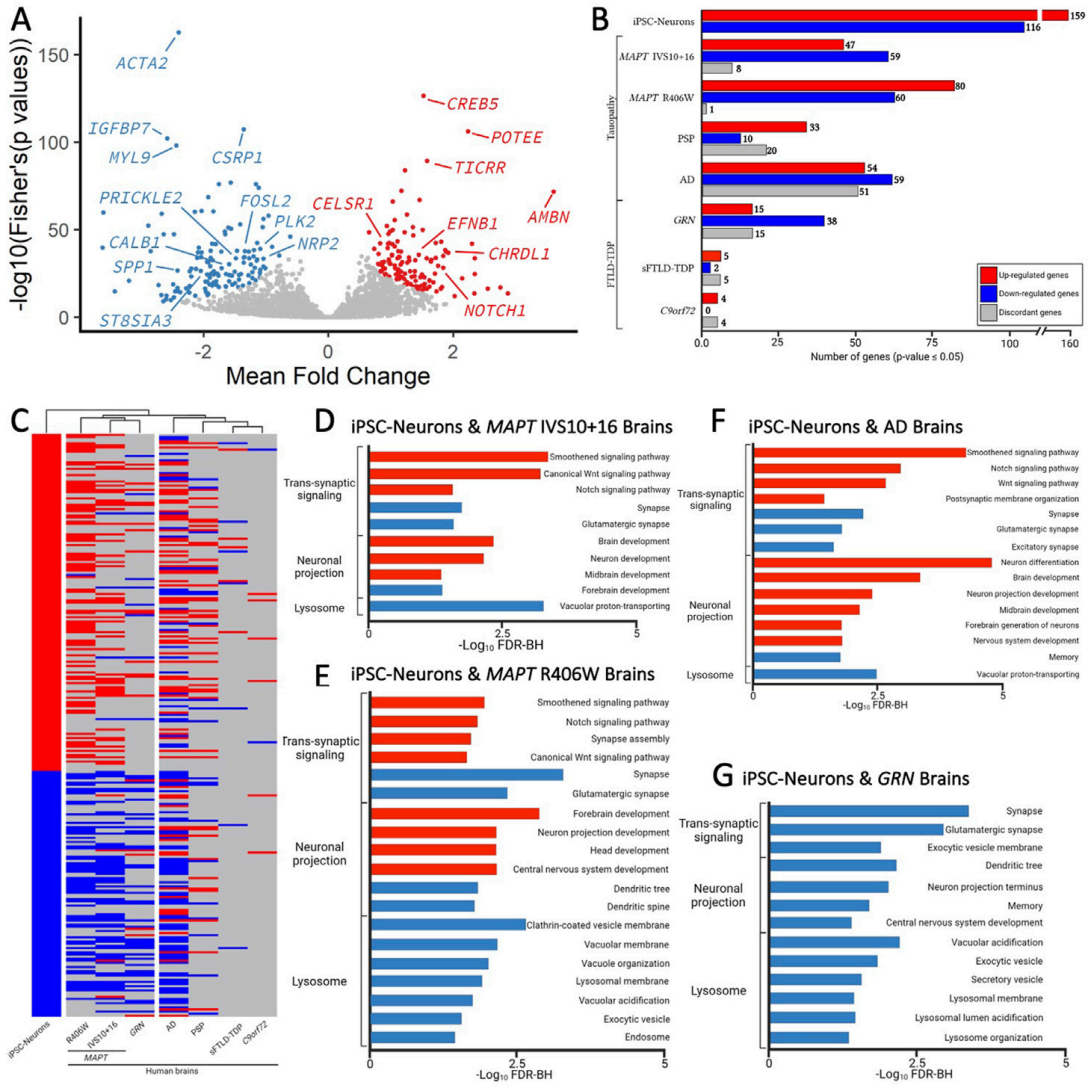
Genes changed in iPSC-neurons from *MAPT* mutations are also altered in brains from tauopathy patients. **(A)** Meta-Volcano plot identifies the common gene expression changes by *MAPT* IVS10 + 16, p.P301L and p.R406W. Red and blue dots represent up- and down-regulated genes, respectively. **(B)** Bar graph of the number of genes differentially expressed in iPSC-neurons from *MAPT* mutations (n = 275) and differentially expressed in brains from tauopathy patients (*MAPT* IVS10 + 16 and p.R406W carriers, PSP, and AD) and from FTLD-TDP (*GRN* mutation carriers, sporadic FTLD-TDP (sFTLD-TDP), and *C9ORF72* expansion carriers). Up-regulated genes, red. Down-regulated genes, blue. Discordant genes (defined as *p* <0.05 and logFold change opposite of iPSC-neurons), gray. **(C)** Heatmap of gene expression of 275 genes in iPSC-neurons and human brains with hierarchical clustering revealing relatedness of cohorts. **(D–F)**. Bar graphs showing the most significant pathways enriched shared between the iPSC-neurons and **(D)** *MAPT* IVS10 + 16 brains; **(E)** *MAPT* p.R406W brains; **(F)** sporadic AD brains; **(G)** *GRN* mutation carrier brains. Pathways from up-regulated genes (red); pathways from down-regulated genes (blue).

To determine the extent to which the iPSC-neuron model recapitulates gene signatures that occur in brains from *MAPT* mutation carriers, we analyzed transcriptomic datasets from *MAPT* IVS10 + 16 and *MAPT* p.R406W carrier brains compared with neuropathology free controls (Jiang et al., 2018). A meta-analysis of the three *MAPT* mutation pairs revealed that there are additional gene expression changes occurring commonly (Figure 5A); thus, for analyses of human brain datasets, we relaxed the *p*-value threshold and examined 275 genes (criteria: *p* <0.05 in single cohort analyses and Fisher’s exact FDR<0.05 in meta-analysis). Of the 275 genes changing in iPSC-neurons, we identified 114 genes in *MAPT* IVS10 + 16 brains and 141 genes in *MAPT* p.R406W brains (Figure 5B; Supplementary Table S12). The majority of the genes that are altered in *MAPT* carrier brains changed in the same direction as the iPSC-neuron model (Figure 5B), making gene signatures from the *MAPT* mutation carrier brains the most similar to the iPSC-neurons in hierarchical clustering analyses (Figure 5C). The genes that change as a function of the *MAPT* mutations in a dish and in disease pathology in the brains are commonly enriched in pathways related to trans-synaptic signaling, neuronal projects, and lysosomal function (Figures 5D, E; Supplemental Tables 13-14).

To determine the extent to which our genetic cellular model of primary tauopathy recapitulates molecular signatures of sporadic tauopathies, we analyzed transcriptomic data from AD, PSP, and control brains (Allen et al., 2016). We found that 63 of the 275 genes were differentially expressed in PSP brains, and 164 genes were differentially expressed in sporadic, late onset AD brains (Figure 5B; Supplementary Table S12). Interestingly, a large number of genes that were implicated in disease processes by the iPSC-neuronal model were changed in opposite directions in the PSP (*n* = 20) and AD brains (*n* = 51; Figure 5B), leading to a similar clustering of these brains with the iPSC-neuronal model but to a lesser extent than what we observe in brains from *MAPT* mutation carriers (Figure 5C). Again, the common genes were enriched in pathways related to trans-synaptic signaling, neuronal projection and lysosomal function (Figure 5F; Supplementary Tables S15–S16).

Synaptic dysfunction, neuronal projections, and lysosomal dysfunction have been implicated in many neurodegenerative diseases. Thus, we asked whether the gene signatures captured in the iPSC-neuronal model represents broader molecular changes in neurodegeneration. To address this question, we examined transcriptomic data from FTLD brains where the primary pathology is TDP-43 pathology (*GRN*, sporadic FTLD-TDP, and *C9ORF72*). There was minimal overlap between the genes changing in a dish as a function of the *MAPT* mutations and genes changing in sporadic FTLD-TDP (*n* = 8) and *C9ORF72* expansion carrier brains (*n* = 12; Figures 5B,C; Supplementary Table S12). Interestingly, we observed substantial overlap between the iPSC-neuronal signatures and those occurring in *GRN* mutation carriers (*n* = 68; Figure 5B; Supplementary Table S12). Surprisingly, this led to brains from *GRN* mutation carriers clustering closely with brains from *MAPT* mutation carriers, appearing to be more similar in expression profile than sporadic tauopathies (Figure 5C). The genes were enriched in lysosomal function but also include trans-synaptic signaling and neuronal projections, all of which are consistent with proposed functions of the progranulin protein (Figure 5G; Supplementary Table S17) (Amin et al., 2022). Together, these analyses identified a set of gene signatures captured in stem cell models of *MAPT* mutations that also change in human brains with genetic and sporadic forms of tau pathology.

## Discussion

**FIGURE 6:**
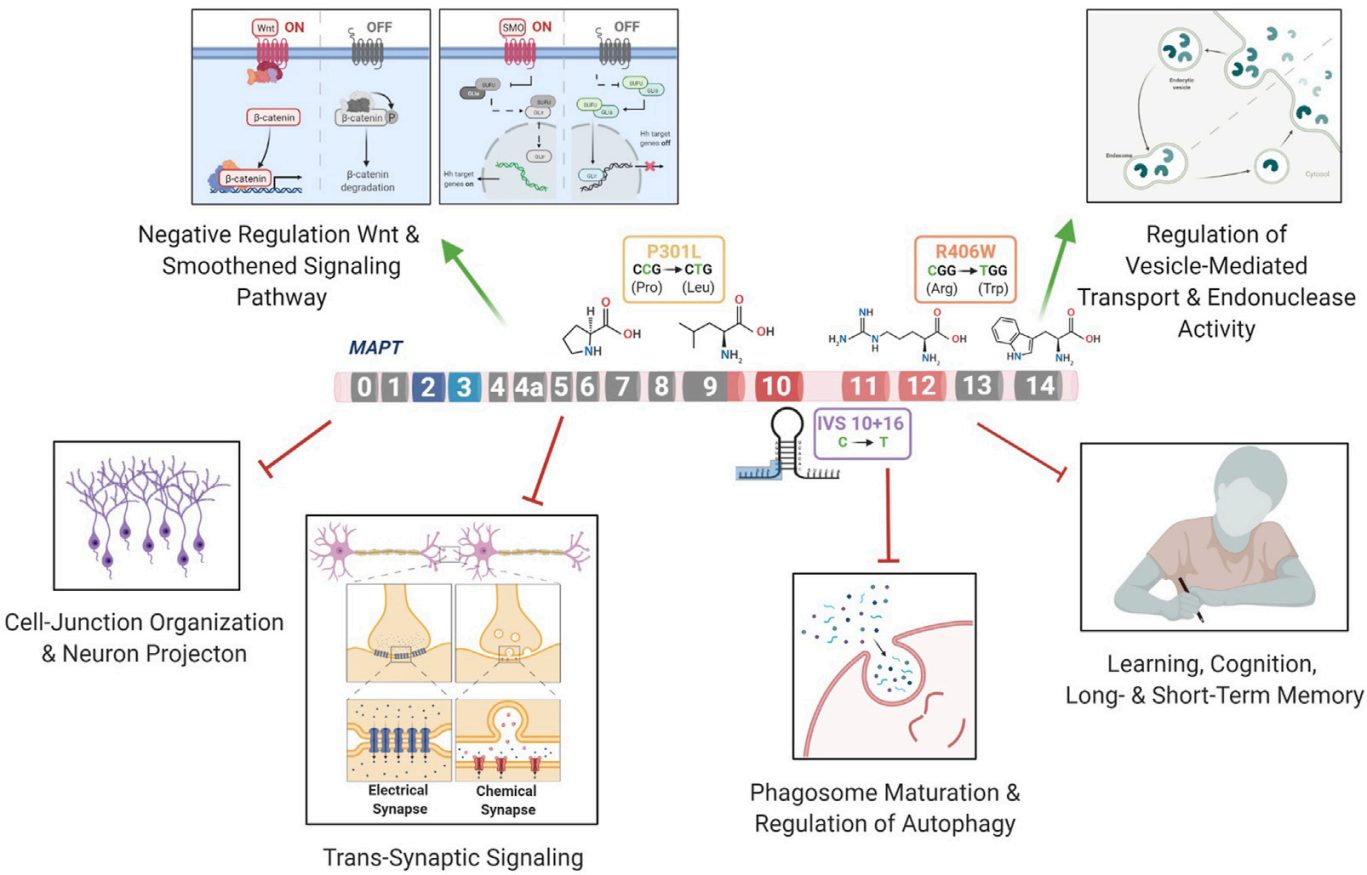
*MAPT* mutations lead to changes in the regulation of pathways related to FTLD-Tau.

The goal of this study was to identify commonly perturbed genes and pathways downstream of *MAPT* mutations and to define a core set of genes that drive disease pathogenesis in FTLD-Tau. *MAPT* mutations result in a range of clinical and neuropathological phenotypes (Whitwell et al., 2009; Ghetti et al., 2015; Moore et al., 2020). Here, we aimed to study three major classes of *MAPT* mutations that represent: splicing mutation (*MAPT* IVS10 + 16), 4R-expressing point mutation (*MAPT* p.P301L) and point mutation expressed in all isoforms (*MAPT* p.R406W). Our results suggest that these three mutations lead to a common series of events, causing the dysregulation of genes associated with pathways involved in synaptic, neuronal, and lysosomal function (Figure 6). Several of the differentially expressed genes were also altered in a mouse model of tauopathy, suggesting that these genes are relevant to disease pathogenesis and tau accumulation. A subset of genes were also found to be dysregulated in human brains from *MAPT* mutation carriers, AD, and PSP donors, illustrating that the molecular signatures we identified in iPSC-neurons are relevant to human disease. Together, this study demonstrates that iPSC-derived neurons capture molecular processes that occur in both mice and human brains, and can be used to model neurodegenerative diseases such as FTLD-Tau.

The three mutations located in distinct regions of the *MAPT* gene produced a common molecular signature of genes that were enriched for pathways involved in trans-synaptic signaling, neuronal projection and lysosomal regulation. Several pathways were related to functional changes described in FTLD. Wnt signaling has been previously shown to be associated with the *MAPT* IVS10 + 16 mutation (Harrison-Uy and Pleasure, 2012) and linked to several neurodegenerative disorders such as AD and FTLD (Korade and Mirnics, 2011; Rosen et al., 2011; Riise et al., 2015; Verheyen et al., 2018; Bottero et al., 2021). In addition, Notch signaling has been shown to be related to the microtubule stability within neurons (Bonini et al., 2013). The Ras signaling regulates basic cellular processes in the construction of neuronal networks, including neurogenesis, vesicular trafficking, or synaptic plasticity (Johnson and Chen, 2012; Qu et al., 2019). The synapse assembly has been associated with Alzheimer’s-type dementia (Clare et al., 2010). Anterograde trans-synaptic signaling; regulation of synaptic; and long-term synaptic potentiation have all been implicated in tauopathies (Purves et al., 2001; Gómez-Palacio-Schjetnan and Escobar, 2013; Liou et al., 2019). Additionally, several genes were enriched for pathways related to the loss of learning or memory and cognition (Rabinovici and Miller, 2010; Bott et al., 2014). Finally, we found that genes associated with dysregulation of the vesicle transport along microtubule pathway; phagosome maturation and autophagy were significantly reduced. This finding is consistent with a recent study of *MAPT* p.V337M-expressing cerebral organoids, where mutant organoids exhibited failure of protein homeostasis, a disruption of autophagy function, and loss of glutamatergic neurons (Bowles et al., 2021). Defects in endolysosomal pathways have been implicated in reduced clearance of protein and cell debris, which may contribute to neurodegeneration (Liu et al., 2012; Viegas et al., 2012; Gunawardena et al., 2014; Etchegaray et al., 2016; Kulkarni and Maday, 2018; Xu et al., 2021c). Thus, here, we identify a series of pathways that are common across different *MAPT* mutation types, suggesting that stem cell models capture defects observed in FTLD-Tau patients (Figure 6).

We report that several genes from the mutant iPSC-neurons were also altered at disease end-stage in the Tau-P301L mouse model of tauopathy: *CALB1, PLK2, CELSR1, CHRDL1, PRICKLE2,* and *ST8SIA3. PLK2* (Polo like kinase 2) is associated with synaptic plasticity and prevention of cell death in neurodegenerative diseases (Kauselmann, 1999; Seeburg et al., 2005; Seeburg et al., 2008; Li et al., 2014; Weston et al., 2021). Modulating the activity of the *PLK2* gene has been proposed as a therapeutic strategy for the treatment of Parkinson’s disease (Oueslati et al., 2013). *CELSR1* (Cadherin EGF LAG seven-pass G-type receptor 1) encodes a receptor protein involved in cell adhesion and receptor-ligand interactions (Hadjantonakis et al., 1997). *CELSR1* has been related to neurodevelopment and maintenance of the nervous system (Boutin et al., 2012), and mutations in this gene have been associated with neural tube defects (Robinson et al., 2012) and AD risk (Patel et al., 2019). *CHRDL1* (Chordin-like 1) plays an important role in CNS development, learning, and promoting synaptic plasticity (Sun et al., 2007; Webb et al., 2012; Gao et al., 2013; Blanco-Suarez et al., 2018). *PRICKLE2* (Prickle planar cell polarity protein 2) is an important cytoplasmic regulator of Wnt/PCP signaling (Katoh, 2005; Barrow, 2006; Vandervorst et al., 2019). Dysregulation of *PRICKLE2* enhances the amyloid β (Aβ) plaque pathology and synaptic dysfunction in mice (Tao et al., 2011; Fujimura and Hatano, 2012). *PRICKLE2* has been proposed to be a potential candidate for the diagnosis and treatment of AD (Sun et al., 2020). *ST8SIA3* (alpha-N-acetyl-neuraminide alpha-2,8-sialyltransferase 3) is involved in neurite growth, cell migration, and synaptic plasticity (Goodman et al., 1997; Lee et al., 1998; Eckhardt et al., 2000; Lin et al., 2019) and plays an important role in the development of Huntington’s disease, schizophrenia, and Parkinson’s disease (Belarbi et al., 2020; Moll et al., 2020). A strong physical interaction was observed between *MAPT* and *CALB1, PLK2, CELSR1, CHRDL1, PRICKLE2,* and *ST8SIA3* and 20 additional genes. The glutamatergic synapse pathway was enriched among these genes. Impairments in glutamatergic circuits predispose GABAergic neurons to dysfunction (Ferrer, 1999; Bowie, 2008; Hughes et al., 2018; Benussi et al., 2019; Murley et al., 2020). Dysregulation of the glutamatergic system has been described in *MAPT* p.V337M cerebral organoids (Borroni et al., 2017; Borroni et al., 2018; Bowles et al., 2021) and *MAPT* p.R406W neurons (Jiang et al., 2018). Thus, our findings suggest that the glutamatergic synapse pathway is disrupted more commonly across *MAPT* mutations.

The absence of overlap in the five remaining genes, and the variability in the expression observed at 4 and 8 months of mouse disease may be driven by several factors. For example, gene expression profiles represent multiple cell-types in the brain which are not included in the iPSC-neuron model, which may mask neuronal-specific gene signatures. Additionally, species differences may also impact the gene signature profile.

*CALB1* was found to be down regulated across the three *MAPT* mutations and reduced at disease end-stage in Tau-P301L mice. The *CALB1* gene (Calbindin 1) regulates the calcium homeostasis in neurons (Noble et al., 2018), which plays a crucial role in neuronal development and memory performance (Sun et al., 2005; Soontornniyomkij et al., 2012; Kook et al., 2014; Goffigan-Holmes et al., 2018; Jung et al., 2020). Given the important role of *CALB1* in neuronal calcium homeostasis, we measured calcium levels in the mutant and isogenic control neurons. We found that calcium levels were significantly reduced in *MAPT* mutant neurons compared with isogenic controls, supporting a dysregulation in calcium homeostasis. Calcium homeostasis is critical for the health and function of neurons and dysregulation of calcium leads to altered synaptic function, endolysosomal function, and neuronal development (Gleichmann and Mattson, 2011). This is in line with recent observations in genetically engineered iPSC expressing the *MAPT* IVS10 + 16 mutation, which showed disturbed intracellular calcium dynamics along with impaired neuronal activity (Britti et al., 2020; Kopach et al., 2021). The Drug-Gene Interaction Database (Freshour et al., 2021) and DrugBank (Wishart et al., 2018) revealed *CALB1* and *PLK2,* also implicated in calcium regulation, are known targets of FDA-approved drugs including tramadol, ethosuximide, levodopa, nicotine and oxcarbazepine, which are currently utilized to treat neurological symptoms (Chen et al., 2015; Tambasco et al., 2018; Morana et al., 2020). Additional work is needed to evaluate calcium across these and other *MAPT* mutations. Thus, restoring calcium homeostasis may be a therapeutically viable approach for treating genetic forms of primary tauopathy.

A subset of the commonly differentially expressed genes from the *MAPT* mutant iPSC-neurons were found to be altered in human brains from *MAPT* IVS10 + 16 and p.R406W carriers. Brains from *MAPT* mutation carriers most closely recapitulated the gene signatures in a dish, but we also detected an overlap among sporadic tauopathies, including AD and PSP. Among the up-regulated genes, we observed an enrichment in pathways involving a negative regulation of: i) nervous system development; ii) neurogenesis; iii) neuron differentiation; iv) neuron projection development; and v) the canonical Wnt signaling pathway. Dysregulation of these pathways have been linked to FTLD patients (Rabinovici and Miller, 2010; Mann and Snowden, 2017; Yousef et al., 2017; Sobue et al., 2018; Bottero et al., 2021). Interestingly, we observed a broad reduction of gene enriched in lysosomal pathways. This is consistent with recent implications of lysosomal dysfunction in genetic and sporadic forms of FTLD-Tau (Polito et al., 2014; Caballero et al., 2018; Silva et al., 2019; Xu et al., 2019; Silva et al., 2020; Xu et al., 2021a; Bowles et al., 2021; Xu et al., 2021b; Caballero et al., 2021; Mahali et al., 2022; Silva et al., 2022). These gene signatures were largely specific for tauopathy; however, we identified a number of lysosomal genes that were altered in iPSC-neurons from *MAPT* mutations and in brains from *GRN* mutation carriers. *GRN* has been implicated in lysosomal function and neuronal integrity (Martens et al., 2012; Kao et al., 2017; Logan et al., 2021; Mohan et al., 2021; Simon et al., 2022). Thus, our stem cell model revealed several genes and pathways that are also altered in primary tauopathy patients.

Our results demonstrate the potential of iPSC technology to investigate disease mechanisms related to FTLD-Tau pathogenesis. A major challenge related to the iPSC technology and neuronal derivatives (iPSC-neurons) when modeling adult-onset neurodegenerative disease concerns capturing age-related phenotypes (Steg et al., 2021). While iPSC-neurons remain relatively immature and do not express the full complement of tau isoforms (Sposito et al., 2015; Sato et al., 2018), we are able to capture molecular signatures that change during disease course in mouse models of tauopathy and patient brains. Thus, there remains tremendous value in a combinatorial multiple model systems approach to identify key pathways that are affected early and remain relevant throughout the disease course.

Furthermore, the observation that different *MAPT* mutations may lead to a common pathophysiological mechanism has not been carefully studied. Thus, these findings have broader implications when considering therapeutic development and trial design. For example, drugs that target these common pathways may be effective across tauopathy patients. This study provides a framework for developing drugs targeting key dysregulated genes.

In conclusion, our approach provides a tractable system to identify genes altered directly by the *MAPT*-IVS10 + 16, p.P301L, and p.R406W mutations, which are relevant to tauopathies and that point to new therapeutic targets. The stem cell lines used in this research allowed for the identification of molecular drivers of disease, which could serve as a platform to identify new targets for drug development. Our iPSC-based cellular models have discovered a common gene signature which is enriched in dysregulated pathways involving synaptic connections, lysosome transport and neuronal development, and mechanisms that have been previously described to be altered in human brains and mouse models of tauopathy.

## Data Availability

The original contributions presented in the study are included in the article/Supplementary Material, further inquiries can be directed to the corresponding author.
