# Supplementary figures and images for: Conserved gene signatures shared among MAPT mutations reveal defects in calcium signaling
Source: Front Mol Biosci. 2023 Feb 9;10:1051494. doi: 10.3389/fmolb.2023.1051494 (PMC9948093; doi:10.3389/fmolb.2023.1051494)

## Supplementary Figure S1

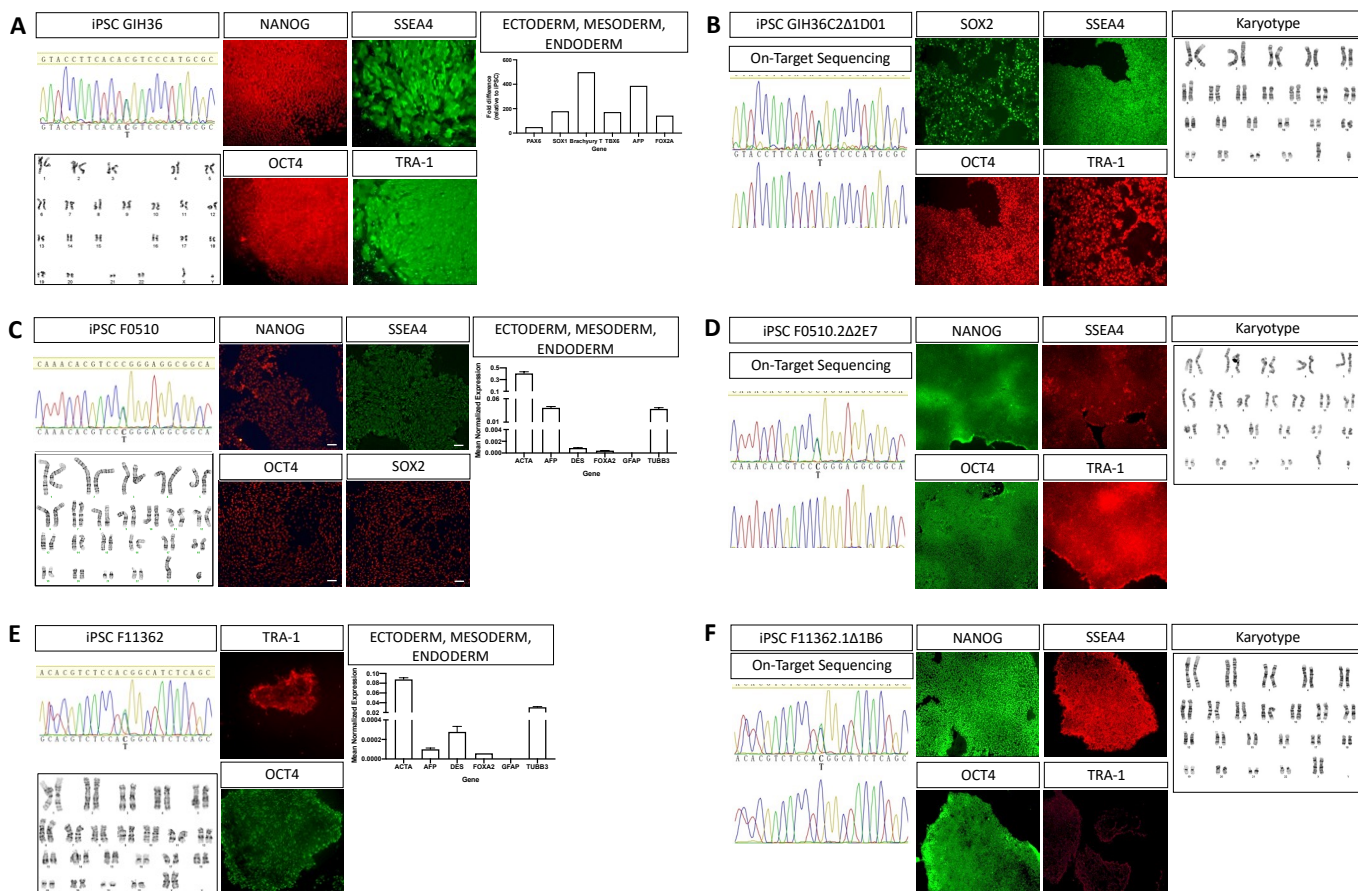

# Supplementary Figure S2

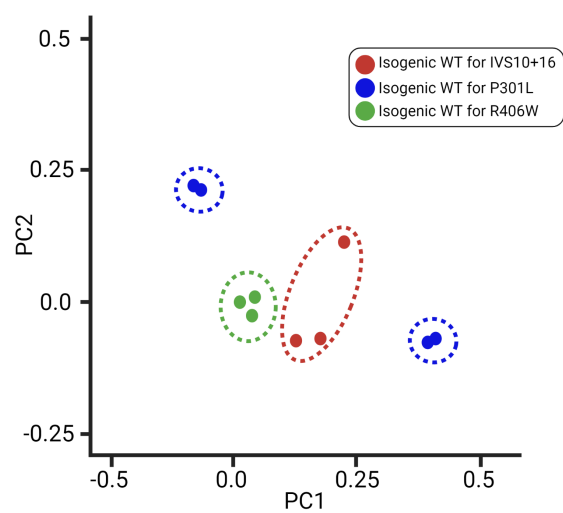

Supplementary Figure S3

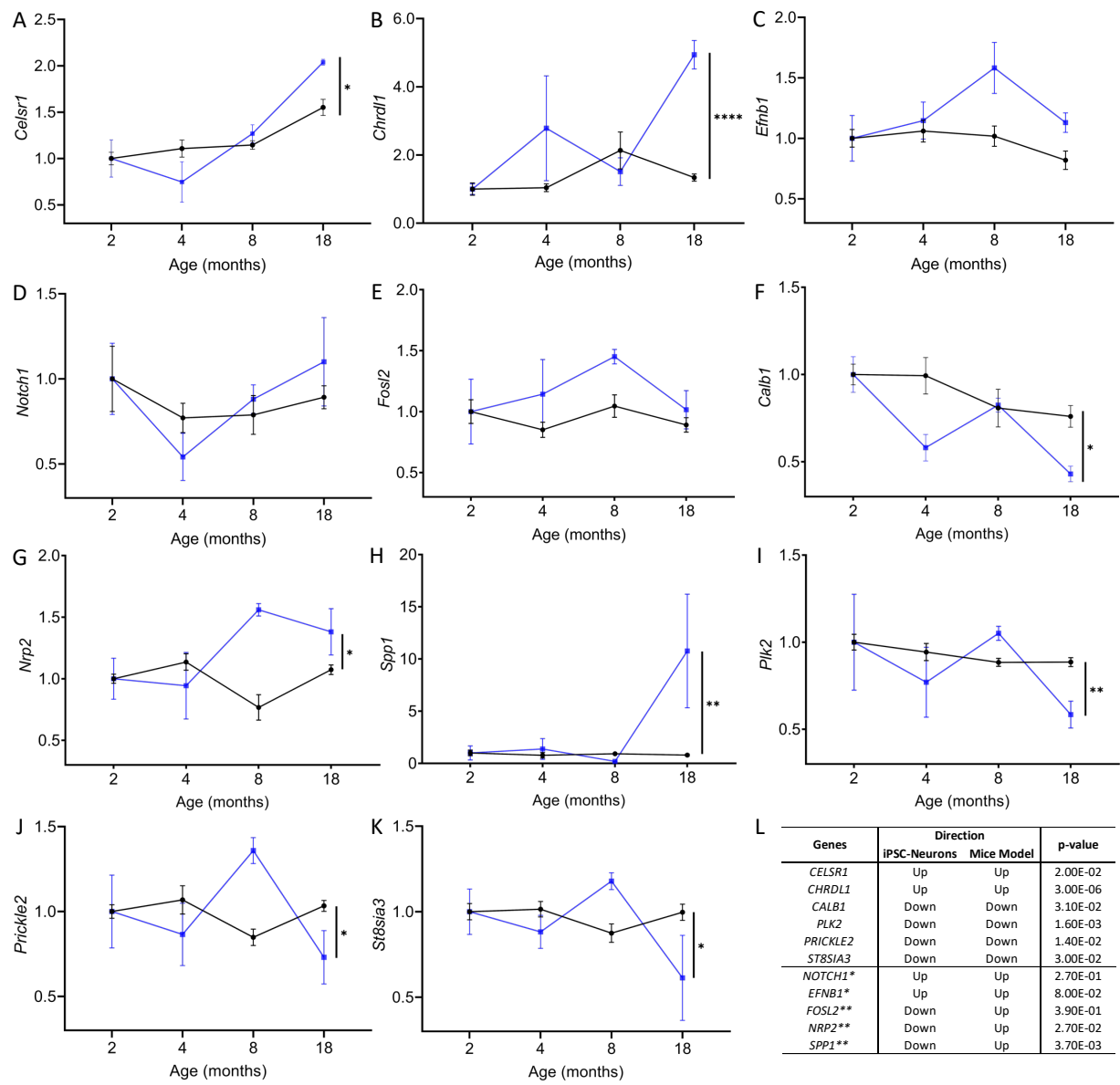

Supplement: Supplementary file 1 [file DataSheet1.pdf]
